# First report of anthelmintic resistance in *Haemonchus contortus* in alpacas in Australia

**DOI:** 10.1186/1756-3305-6-243

**Published:** 2013-08-22

**Authors:** Abdul Jabbar, Angus JD Campbell, Jennifer A Charles, Robin B Gasser

**Affiliations:** 1 Faculty of Veterinary Science, The University of Melbourne, Werribee, VIC 3030, Australia

**Keywords:** Anthelmintic resistance, Alpaca, Macrocyclic lactones, Gastrointestinal nematodes, *Haemonchus contortus*

## Abstract

**Background:**

Parasitic nematodes can cause substantial clinical and subclinical problems in alpacas and anthelmintics are regularly used to control parasitic nematodes in alpacas. Although anthelmintic resistance has been reported in ruminants worldwide, very little is known about anthelmintic resistance in alpacas. The present study was carried out to confirm a suspected case of anthelmintic resistance in *Haemonchus contortus* in alpacas in Australia.

**Methods:**

Post mortem examination of an alpaca was conducted to determine the cause of its death. To confirm a suspected case of macrocyclic lactone (ML) resistance in *H. contortus* in alpacas, a faecal egg count reduction test (FECRT) was performed using closantel (7.5 mg/kg) and ivermectin (0.2 mg/kg). Nematode species were identified by morphological and molecular methods.

**Results:**

Post mortem examination of a 1-year-old female alpaca that had died following a brief period of lethargy, anorexia and recumbency revealed severe anaemia, hypoproteinaemia and gastric parasitism by adult *Haemonchus contortus*, despite recent abamectin (0.2 mg/kg) treatment. Based on these findings and the exclusive use of MLs in the herd over the preceding six years, ML resistance in parasitic nematodes of alpacas on this farm was suspected. FECRT revealed that the efficacy of closantel was 99% (95% CI 93-100), whereas that of ivermectin was 35% (95% CI 0-78), indicating that the treatment failure was likely due to the presence of ML-resistant nematodes. Larval culture of faecal samples collected following ivermectin treatment consisted of 99% *H. contortus* and 1% *Cooperia oncophora*, a result confirmed using a PCR assay.

**Conclusions:**

This study provides the first evidence of ML resistance in *H. contortus* in alpacas in Australia. Based on the extent of anthelmintic resistance in sheep gastrointestinal nematodes in Australia, veterinarians and alpaca owners should be encouraged to implement integrated parasite management strategies to improve nematode control in alpacas.

## Background

In the last two decades, domesticated South American Camelids (SACs), particularly llamas (*Lama glama*) and alpacas (*Lama pacos*), have been farmed increasingly for their fine fleece and adaptability to many climatic regions. Smaller numbers are also kept as pets on ‘hobby’ farms [1]. There are now in excess of 90,000 alpacas registered in Australia, with more than 90% of these being of the Huacaya breed [2].

In intensive farming contexts, parasitic nematodes can cause substantial clinical and subclinical problems in SACs, leading to economic loss from suboptimal production of fibre, meat and/or leather [3]. Domesticated alpacas often share pasture with other livestock species such as sheep, and are grazed under more intensive grazing conditions than in their native countries, factors which significantly increase the probability of their acquiring nematode infections [3-6]. Farmers regularly administer various classes of anthelmintics to alpacas to control nematodes. Although anthelmintic resistance has been reported in ruminants worldwide, very little is known about anthelmintic resistance in SACs [7-9]. Here, we investigated suspected macrocyclic lactone (ML) resistance in *Haemonchus contortus* on an alpaca farm in Australia, following several deaths of mature alpacas in a herd in which MLs (including abamectin) had been used regularly.

## Methods

### Herd description

The investigation was carried out on a farm with a herd of 115 alpacas in the Yarra Valley, north-east of Melbourne (37°39′22″S 145°30′50″E), Victoria. This region has an average annual rainfall of 1170 mm, with mild summers and cool winters. Alpacas in the herd were routinely vaccinated against clostridial diseases (caused by *Clostridium perfringens* type D, *C. tetani*, *C. novyi* type B, *C. septicum* and *C. chauvoei*) and were drenched orally with macrocyclic lactone (ML) anthelmintics, once each summer and once each winter. Abamectin had been used in the previous two years and moxidectin for four years prior to that. No other anthelmintic classes had been used on the farm in recent years. Replacement alpacas were bred on the farm or acquired from other parts of Australia. Alpacas introduced into the herd were treated with oral abamectin (0.2 mg/kg) and kept in isolation for one week. No other species of livestock were kept on the farm.

### Case history

The present investigation was instigated in September 2012 after the deaths of eight adult alpacas in the herd over the preceding five months. Five animals had died in late autumn (April and May) and three others in early spring (September). Affected animals were usually in good condition but became lethargic, weak and recumbent, and thence died in one to two days, despite treatment with oral abamectin (0.2 mg/kg) and various injectable antibiotics. Field post mortem examinations conducted by a local veterinarian revealed that all of the affected animals were anaemic. The carcase of a ninth animal, 1-year-old female Huacaya alpaca weighing 47 kg, was submitted to the Veterinary Clinical Centre, The University of Melbourne for post mortem examination.

Based on post mortem findings of severe anaemia, hypoproteinaemia and gastric parasitism by adult *H. contortus*, and the drenching history of the herd, we suspected a problem associated with ML resistance.

### Faecal egg reduction test

A faecal egg count reduction test (FECRT) was performed on the farm, according to the World Association for the Advancement of Veterinary Parasitology (WAAVP) guidelines for evaluation of anthelmintic efficacy in ruminants [10]. In an attempt to prevent further deaths of alpacas, the farmer had treated most animals on the farm with Closicare with Selenium® (closantel 37.5 g/L, selenium 0.5 g/L; Virbac Animal Health Pty Ltd, New South Wales (NSW), Australia) at a dose rate of 1 mL/5 kg body weight, so that only 23 adult female alpacas were available for the FECRT. These animals were randomly allocated to three groups for treatment with Ivomec Liquid for Sheep® (ivermectin 0.8 g/L; Merial Pty Ltd, NSW, Australia) or Closicare with Selenium or as a control group: Group 1 – 8 animals, Ivomec at 1 mL/4 kg body weight; Group 2 – 8 animals, Closicare with Selenium at 1 mL/5 kg; Group 3 – 7 animals, untreated control. The anthelmintics were administered orally at the dose for the heaviest animal in the group. Individual faecal samples were collected from the rectum prior to treatment (day 0) and 11 days after treatment. Faecal egg counts of both thick-shelled strongylid and *Nematodirus* eggs were determined using a modified McMaster technique [11], with a sensitivity of 30 eggs per gram of faeces (EPG). The reduction in EPG was calculated using the program RESO FECRT v4.0 (http://www.vetsci.usyd.edu.au/sheepwormcontrol/index.html). A population of strongylid nematodes was defined as resistant to an anthelmintic if the reduction in EPG was less than 95% and the lower 95% confidence limit of the percentage of reduction was less than 90% [12].

### Identification of nematode species

Standard faecal larval cultures were performed for each of the three groups in the FECRT, using a 20 g composite of individual faecal samples incubated at 25°C for 10 days [13].

In addition, we used a polymerase chain reaction (PCR)-coupled method [14] for the unequivocal identification of trichostrongylid eggs present in alpaca faeces on the farm under investigation. In brief, genomic DNAs from strongylid eggs, isolated from pooled faecal samples from each group, were extracted using PowerSoil® DNA Isolation Kit (MO BIO Labs, Inc., California, USA) and specifically tested for *Chabertia ovina*, *Cooperia oncophora*, *H. contortus*, *Oesophagostomum columbianum, O. venulosum*, *Teladorsagia circumcincta* and *Trichostrongylus* spp. [14]. Negative (no DNA) and known positive controls were included in each set of PCRs. Amplicons were subjected to 1.5% agarose gel electrophoresis, automated DNA sequencing (BigDye® Terminator v.3.1 chemistry, Applied Biosystems, Foster City, California, USA), and BLASTn analysis (http://blast.ncbi.nlm.nih.gov) to establish the ‘top hits’ to all nucleotide sequences available in current databases.

## Results

### Post mortem examination

**Figure 1:**
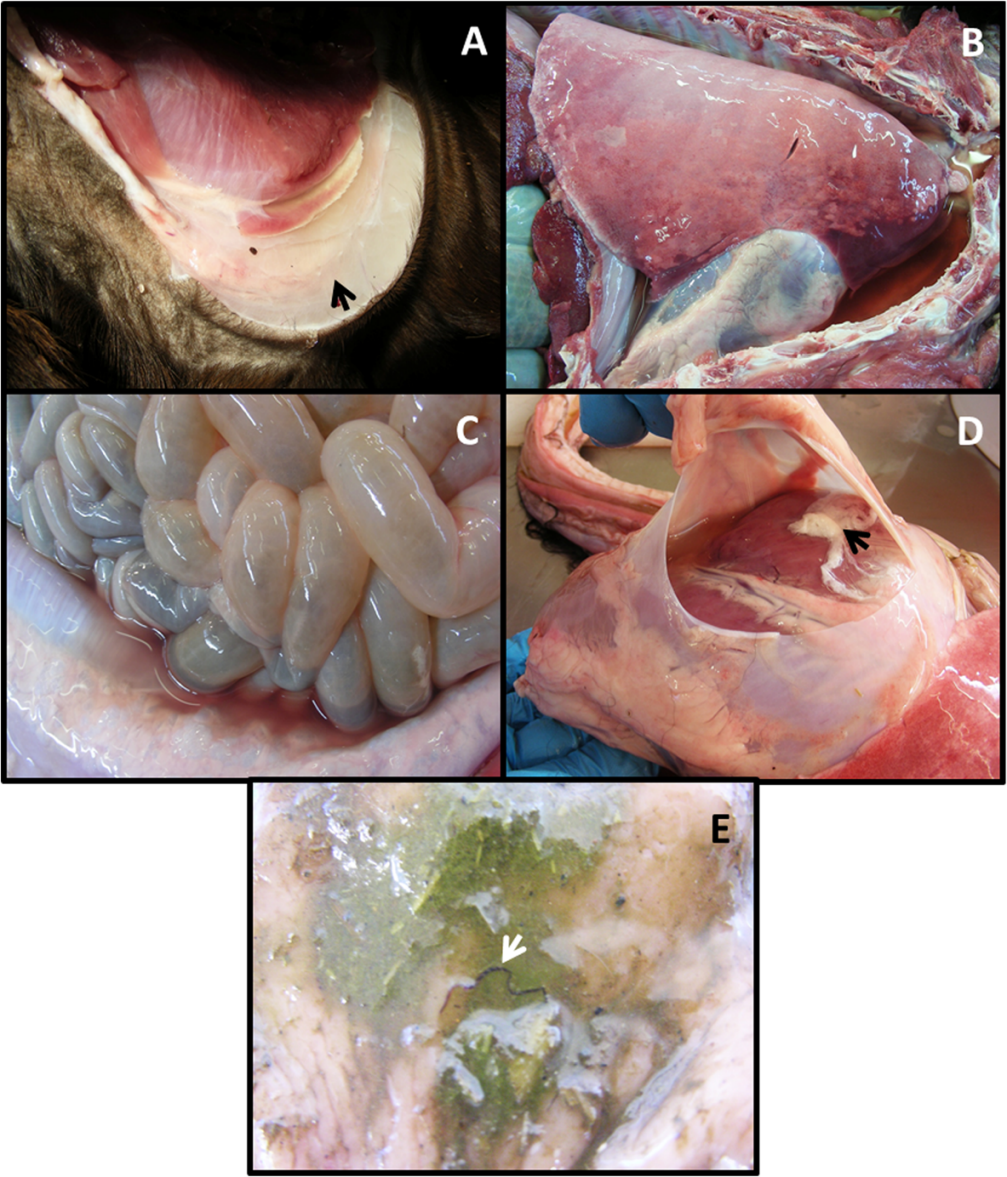
**Lesions in an alpaca that died of chronic haemonchosis. A**, severe subcutaneous oedema over a stifle joint; **B**, hydrothorax with compression atelectasis of the cranioventral lungs and diffuse pulmonary oedema; **C**, serosanguineous ascites; **D**, hydropericardium with a fresh fibrin coagulum; **E**, adult *Haemonchus contortus* on the mucosal surface of the third compartment of the stomach.

A 1-year-old female Huacaya submitted for post mortem examination had been sick for several weeks and had received oral abamectin at the sheep dose rate of 0.2 mg/kg, one week before its death. Post mortem findings included severe pallor of the oral and conjunctival mucosae, skeletal musculature, liver and kidneys and remarkably watery blood within the heart and major blood vessels. These changes were consistent with severe anaemia. There was mild submandibular oedema and severe subcutaneous oedema (up to a 4 cm depth) in dependent areas along the ventral trunk and radiating down the proximal fore and hind limbs (Figure 1A). Moderately severe hydrothorax, hydropericardium and ascites were also present (Figure 1B-D) and there was moderately severe diffuse pulmonary oedema and mild oedematous distension of the soft tissues of the larynx. The oedema was suspected to be referable to hypoalbuminaemia. However, determination of an accurate plasma protein concentration and a packed cell volume could not be undertaken due to post mortem lysis of erythrocytes. A small number of adult nematodes were observed on the mucosal surface of the third compartment of the stomach (Figure 1E). A total worm count revealed *H. contortus* (*n* = 460) in this gastric compartment but no worms were found in the duodenum and proximal jejunum. An egg count performed on a sample of rectal faeces revealed 6,600 thick-shelled strongylid eggs per gram. Histological evaluation of the viscera revealed no other significant disease process and the cause of death was concluded to be severe haemorrhagic anaemia and hypoproteinaemia referable to the gastric nematode infestation.

### Faecal egg count reduction test and species identification

Analysis of day 0 FECs revealed that the animals in the ivermectin group had low egg counts. As no other untreated animals were available on the farm, the control animals were treated with ivermectin on day 11 and faecal samples were collected 15 days later (day 26). The efficacy of ivermectin was calculated as the percentage change in mean FEC between day 11 and day 26. The results of the FECRT performed on the alpaca farm are presented in Table 1. The efficacies of closantel and ivermectin were 99% (95% confidence interval (CI): 93-100%) and 35% (95% CI: 0-78%), respectively.

**Table 1:** Faecal egg count reduction test on an alpaca farm in Victoria, Australia

**Anthelmintic treatment**	**Mean EPG**		FECR% (95% CI*)
	**Day 0**	**Day 14**	
Control	797 ± 798	434 ± 419	-
Closantel	518 ± 738	5 ± 11	99% (93–100%)
Ivermectin	434 ± 419	283 ± 264	35% (0–78%)

Faecal egg counts (eggs per gram (EPG) ± standard deviation) and percentage faecal egg count reduction (FECR%) in alpacas treated with two anthelmintics.

*CI = confidence internal.

Faecal larval cultures revealed that 82% of the larvae from the pre-treatment samples were *H. contortus*, with smaller percentages of *C. oncophora* (11%), *Nematodirus* spp. (2.5%) and *Trichostrongylus* spp. (4.5%). After treatment with ivermectin, 99% of the larvae in the faecal sample were *H. contortus* and 1% were *Cooperia oncophora*. Small numbers of *H. contortus, C. oncophora*, *Nematodirus* spp. and *Trichostrongylus* spp. larvae were recovered from a pooled faecal culture from alpacas treated with closantel, although the number was insufficient to reliably calculate the species proportions in the sample.

Genus-specific PCR from eggs from pooled faeces from each group followed by the sequencing of amplicons and analysis of the ITS-2 sequence data revealed the presence of *C. onchophora, H. contortus* and *T. axei*, based on comparisons with corresponding well-defined ITS-2 reference sequences [GenBank accession numbers JN128897, AJ544463 and AY439026 for *H. contortus*, *C. onchophora* and *T. axei*, respectively] available in the GenBank database.

## Discussion

Resistance in major gastrointestinal nematodes (GINs) of sheep against all major classes of anthelmintics is well documented in Australia [15-17]. However, almost nothing is known about the susceptibility of alpaca GINs to anthelmintics and the widespread use of a limited number of compounds to control GIN in alpacas increases the risk of development of anthelmintic resistance. Our report is the first documented evidence of anthelmintic resistance in alpacas in Australia, and follows reported anthelmintic treatment failures in SACs in the USA [8] and Belgium [9]. Oral and injectable MLs are very widely used in alpacas in Australia to control GINs (J. Vaughan, pers. comm.).

On the farm under study, morbidity and mortality of alpacas over several months were suspected to be referable to haemonchosis, despite long-term regular oral drenching of all animals with abamectin and moxidectin. The history and the post mortem findings strongly suggested ML resistance had led to a failure of worm control on this farm. This suspicion was confirmed by the FECRT, which indicated that ivermectin was only 35% effective, with this inferred resistance largely confined to *H. contortus*. Such poor efficacy of ivermectin would mean that the MLs used on the farm would have also been ineffective in controlling other nematode (*Cooperia* spp. and *Trichostrongylus* spp.) infections. Had ante mortem FEC been performed on the moribund alpaca on this farm, a FEC of 6,600 EPG in an animal drenched regularly with MLs should have raised suspicions of a failure of worm control, even before the FECRT was conducted.

A number of factors might have contributed to the development of ML resistance on this farm, including those known to be associated with the development of anthelmintic resistance in small ruminants [18]. Firstly, MLs had been used exclusively on the property for at least the last six years, and the only variation in anthelmintic was between different types of MLs. Without the use of other classes of anthelmintics to remove ML-resistant worms, it could be expected that the prevalence of ML-resistant worms on the farm would steadily increase over time. The selection pressure for drench resistance in *H. contortus* may have also been increased by the ecology of this species in this environment, since *Haemonchus* larvae survive poorly on pasture during cool winters [19], leaving a small population of ‘refugia’ from the winter treatments [20]. Secondly, the pharmacokinetic properties of MLs in alpacas are not well described, and inappropriate dosing may have contributed to the development of ML resistance. No anthelmintics are registered for use in alpacas in Australia, and the animals on this farm received oral doses at sheep dose rates, although some authors suggest that alpacas should receive 1.5 times the dose rate of MLs for sheep [8,21]. Off-label anthelmintic use is further complicated by the observation that different formulations of orally administered compounds affect absorption in SACs [22] and those other anthelmintic classes require even greater dose rates than those used in sheep [23]. Scales were not available on the farm, and under-dosing of anthelmintics might also have contributed to the development of anthelmintic resistance on this farm. Our findings, along with previous studies, highlight the need for determining the correct therapeutic doses of various anthelmintics in SACs.

Although these factors might explain how the observed anthelmintic resistance developed, an alternative proposal is that drench-resistant worms were introduced with alpacas bought in from other farms [24,25]. Such a drench-resistant worm population could have arisen in alpacas on another property or developed in other co-grazing ruminant species with subsequent cross-infection of alpacas before they were moved to the current farm [3,26-28]. Frequent movement of alpacas amongst different farms represents an important potential route of dissemination of resistance.

Despite the apparent failure of MLs on the present farm and the suspected death of multiple alpacas from haemonchosis, it is interesting to note that few tested animals had low packed cell volumes at the time of the FECRT (data not shown). This may have reflected individual differences in susceptibility to parasitism [29], with the most heavily parasitised animals all dying before we commenced our investigation. Alternatively, different grazing management of groups of animals on the farm may have affected pasture contamination with nematode larvae or led to varying rates of infection amongst different groups. Nonetheless, it is important to note that most animals on the farm appeared to be clinically unaffected despite the poor drench efficacy and disease in their herd mates. Effective mechanisms are required for monitoring SACs to identify the need for strategic and/or tactical anthelmintic treatment, such as the Faffa Malan's Chart (FAMACHA^©^ system) used in sheep and goats [30].

Most larvae in the post-treatment faecal cultures from the alpacas treated with ivermectin were *H. contortus*, although a small percentage of *C. oncophora* (1%) was also present. ML resistance occurs in *Cooperia* spp. in cattle in Australia [31], although it is difficult to assess the importance of our observation in the present study.

## Conclusions

This study provides the first evidence of ML resistance in *H. contortus* in alpacas in Australia. Based on the extent of anthelmintic resistance in sheep GINs in Australia, veterinarians and alpaca owners should be encouraged to implement integrated parasite management strategies to improve nematode control in alpacas and manage anthelmintic resistance to avoid both fulminant disease and subclinical parasitism.

## Abbreviations

CI: Confidence interval; EPG: Eggs per gram of faeces; FAMACHA: Faffa Malan's Chart; FECRT: Faecal egg count reduction test; GIN: Gastrointestinal nematodes; ML: Macrocyclic lactone; NSW: New South Wales; PCR: Polymerase chain reaction; WAAVP: World Association for the Advancement of Veterinary Parasitology.

## Competing interests

The authors declare that they have no competing interests.

## Authors’ contributions

AJ, AJDC and RBG conceived the project; AJ and AJDC undertook the field experiments; AJ carried out laboratory work; JAC conducted the post mortem examination; AJ, AJDC, and JAC carried out data analysis and interpretation; AJ wrote the draft manuscript with critical input from AJDC, JAC and RBG. All authors read and approved the final manuscript.
